# Polystyrene nanoplastics inhibit reproduction and induce abnormal embryonic development in the freshwater crustacean *Daphnia galeata*

**DOI:** 10.1038/s41598-017-12299-2

**Published:** 2017-09-21

**Authors:** Rongxue Cui, Shin Woong Kim, Youn-Joo An

**Affiliations:** Department of Environmental Health Science, Konkuk University, 120 Neungdong-ro, Gwangjin-gu, Seoul, 05029 Korea

## Abstract

We assayed the toxicity of polystyrene nanoparticles (PS-NP, 52 nm) to *Daphnia galeata*. Survival and reproduction were significantly decreased in individuals exposed to 5 mg/L of PS-NP for 5 days, and embryos showed abnormal development, including a low hatching rate. Using fluorescence confocal microscopy, we recorded the transfer of PS-NP from the external surface of the body to the internal organs, including the thoracic appendices, ovaries, caudal appendices, and brood chamber, as well as PS-NP storage in lipid droplets. Although embryos were exposed to PS-NP in the brood chamber, they did not internalize PS-NP. Exposed *D*. *galeata* adults that were not pregnant stored significantly fewer lipid droplets than did the control group, and the lipid droplets that they did store were smaller; meanwhile, there were no significant changes in lipid storage in exposed pregnant individuals. Some embryos showed a high level of lipid storage, a response that occurs when embryos experience an abnormal state, and these embryos showed a very low hatching rate. However, the offspring of exposed adults showed normal survival and lipid storage. This study provides visual evidence that confirms the transfer and effects of PS-NP in *Daphnia* species, and suggests a relationship between toxicity and lipid storage.

## Introduction

A large amount of plastic is produced and discarded into the environment every year, causing global environmental problems^1^. Plastic slowly degrades into micro- (<5 mm) and nano-sized (<100 nm) particles owing to physical, chemical, and biological processes^2–4^. These particles are largely detected in marine systems^1,5^, and are of great concern because of their abundance and adverse effects on living organisms^6–9^. Plastic particles, however, also accumulate in soil and freshwater environments^10,11^. Many previous freshwater studies have reported on the vast distribution of micro-sized plastics, e.g., 0–466,305 particles/km^2^, 100 particles/km^2^, and 20,264 (997–44,435) particles/km^2^ in a USA lake^12^, UK estuary^13^ and Mongolia lake^14^, respectively. The effects of plastics on freshwater organisms are, however, still largely unknown^15,16^. Recent studies have reported various toxicities of micro- and nano-sized plastics on freshwater organisms, including green algae^17^, rotifers^18^, zooplankton^19–23^, and fish^24–27^. Particles have been found in the digestive organs of *Brachionus koreanus* ^18^, the gastrointestinal tract of *Daphnia* species^21,23^, and the gill and gut of zebrafish^26^. Nano and micro-sized plastics are reported to cause adverse effects such as reduced survival^21^, immobilization, abnormal behavior^22^, and changes in feeding activity^21^ of adults during acute test periods, as well as reduced survival, reproduction, body size, and abnormal physical formation of the subsequent generation^19^. Various mechanisms are responsible for histopathological changes^26,28^, oxidative stress^18^, and alteration of lipid metabolism^29,30^ as a result of exposure to plastic particles. However, we lack a complete understanding of the behavior of plastic particles in biological systems, and, therefore, also a complete understanding of their effects.

In the present study, we investigated polystyrene nanoparticle (PS-NP, 52 nm; Supplementary Fig. S1) toxicity in the freshwater crustacean *Daphnia galeata*. This species was chosen for PS-NP toxicity evaluation because it is one of the freshwater standard test species that is suggested in several international test guidelines^31^, it is a nonselective filter-feeder^32^, and it is widely distributed in the northern hemisphere freshwater^33^. We visually confirmed the transfer of PS-NP from the media, Moderately Hard Water (MHW), to the internal organs of *D*. *galeata*, including the intestine, ovary, and brood chamber, and to developing embryos within the brood chamber. The changes in lipid storage were measured using a quantitative analysis of lipid droplets in adult *D. galeata*. An embryonic development assay was also conducted and the effects on the offspring of exposed adults were examined.

## Results

### PS-NP effects on *D. galeata* adults and embryo developments

**Figure 1:**
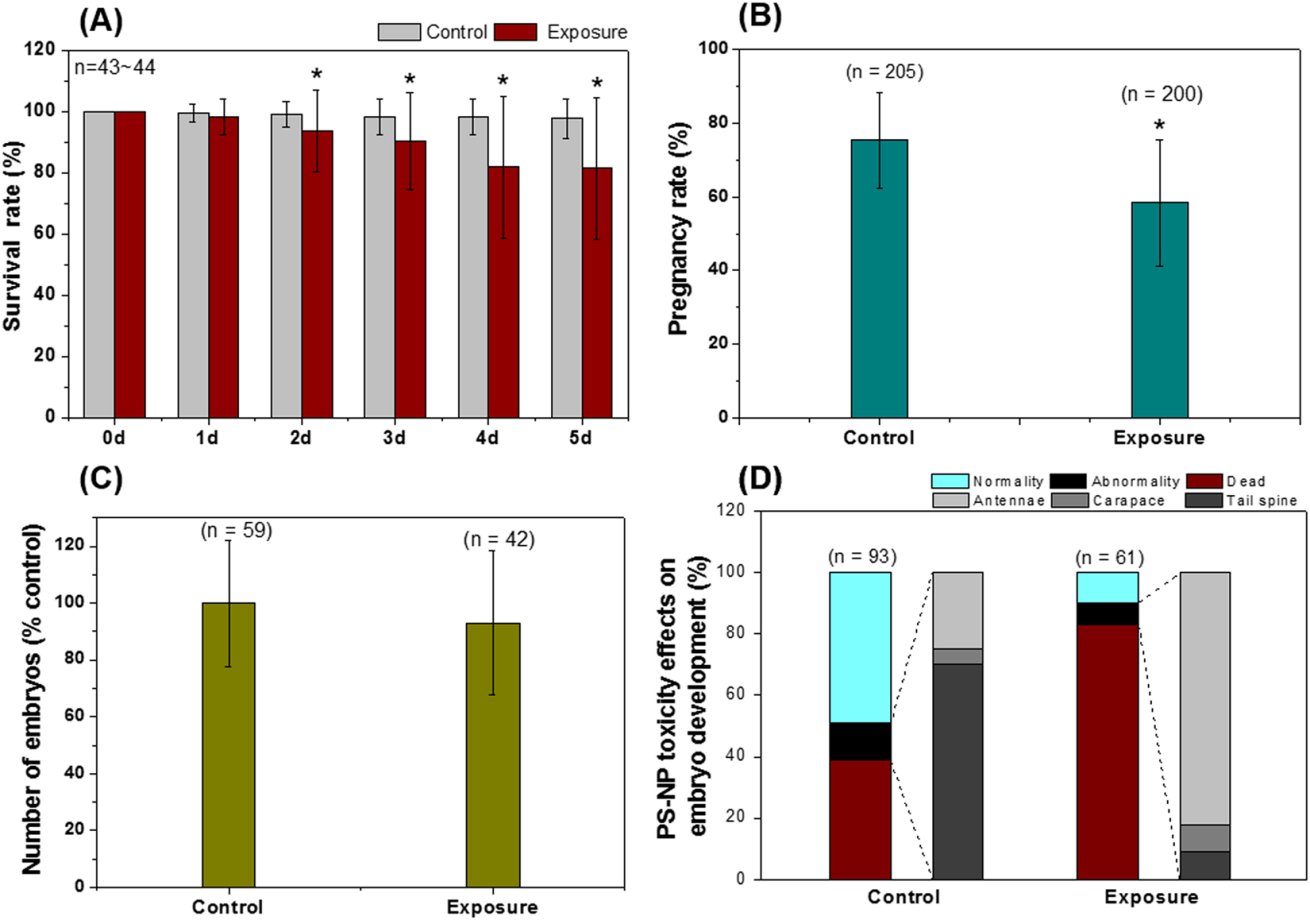
Effects of 5 days of exposure to 5 mg/L polystyrene nanoparticles (PS-NP) on the (**A**) survival rate, (**B**) pregnancy rate, and (**C**) number of embryos in *Daphnia galeata* adults. (**D**) Number of normal, abnormal, and dead embryos isolated from the parent generation. Although abnormalities were also observed in the post-abdominal claw, Malpighian tubule and sensory bristles, only the percentage abnormalities of the antennae, carapace, and tail spine are shown here. Asterisk (*) indicates a significant difference (*p* < 0.05) compared with the control group.

The survival of *D. galeata* exposed to PS-NP (5 mg/L) was observed daily for 5 days. The survival rates significantly decreased (*p* < 0.05) after 2 days of exposure until the end of the study (day 5) (Fig. 1A). At the end of test (day 5), significantly fewer individuals were pregnant in the exposed group (58 ± 17%, *p* < 0.05) than in the control group (75 ± 13%) (Fig. 1B). There were 5 ± 2 embryos per pregnant individual in both the control and exposed groups (Fig. 1C). Embryos were isolated from parent survivors, and abnormalities were observed during their development. Only 10 ± 15% of exposed embryos were considered normal, and there was an 83 ± 25% mortality of the exposed embryos (Fig. 1D).

### Biodistribution of PS-NP in *D*. *galeata*

**Figure 2:**
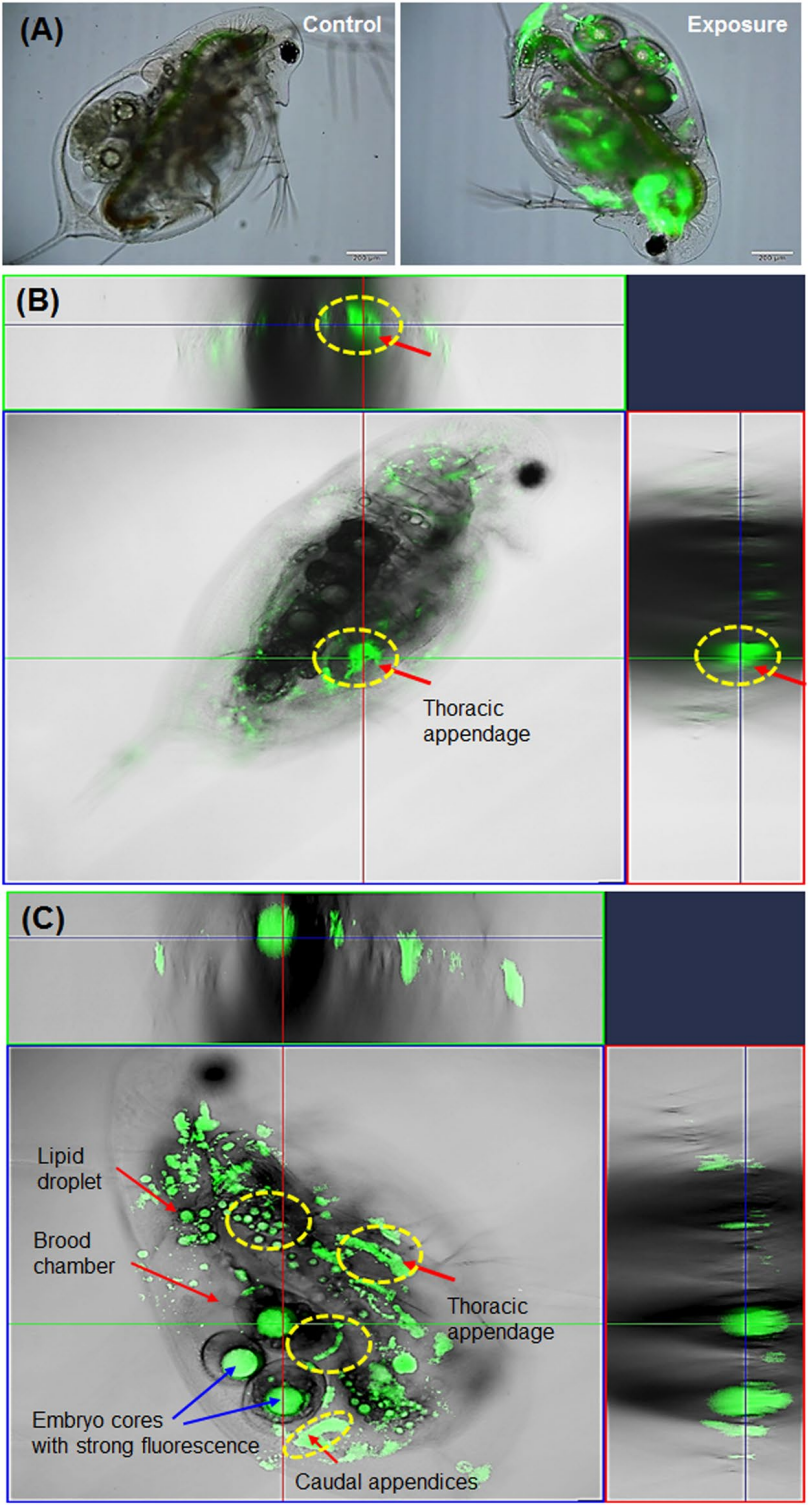
(**A**) Optical microscopic images of *Daphnia galeata* from control and exposed groups. (**B**,**C**) Confocal laser scanning microscopic images of *D. galeata* exposed to polystyrene nanoparticles (PS-NP) for 5 days. Confocal images are obtained from cross-sectional analysis by Z-stack techniques, and the upper and right side show the x-z-axis and y-z-axis, respectively. Red arrows indicate the organs of *D. galeata*, and blue arrows indicate the embryo core with strong green fluorescence. Yellow circles mark the biodistribution of PS-NP.

Compared to the control group, the PS-NP exposed individuals showed a strong green fluorescence throughout their bodies (Fig. 2A). Z-stack images (with a 1-μm depth) of *D. galeata* were obtained with *x*-*z* and *y*-*z* scales at the top and right, respectively. These interior images established that PS-NP were highly distributed on the thoracic appendices (Fig. 2B), and that the green fluorescent particles were clearly observed in the brood chamber, ovary, and embryos (Fig. 2C). The PS-NP were attached to embryos in the brood chamber, and some particles were blended with lipid droplets. Interestingly, we also found aggregations of PS-NP on caudal appendices, an important organ involved in protecting embryos against foreign substances. The results of the visual 2.5D analysis showed morphological images of thoracic appendages, ovary, oocytes, brood chamber, and lipid droplets (Supplementary Fig. S2A), and green fluorescence distributed in these organs (Supplementary Fig. S2B). The potential pathways of PS-NP intake are shown in Fig. S3 (Supplementary Information). The behavior and transfer in *Daphnia* species depends on the following: (a) PS-NP transfer into the intestine through the mouth (Supplementary Fig. S4A); (b) PS-NP adsorption during the filtration process of the thoracic appendices (Supplementary Fig. S4B); (c) PS-NP penetration into the brood chamber thorough the caudal appendices (Supplementary Fig. S4C); and (d) PS-NP adsorption on the surface of the organism, including the antennae (Supplementary Fig. S4D).

**Figure 3:**
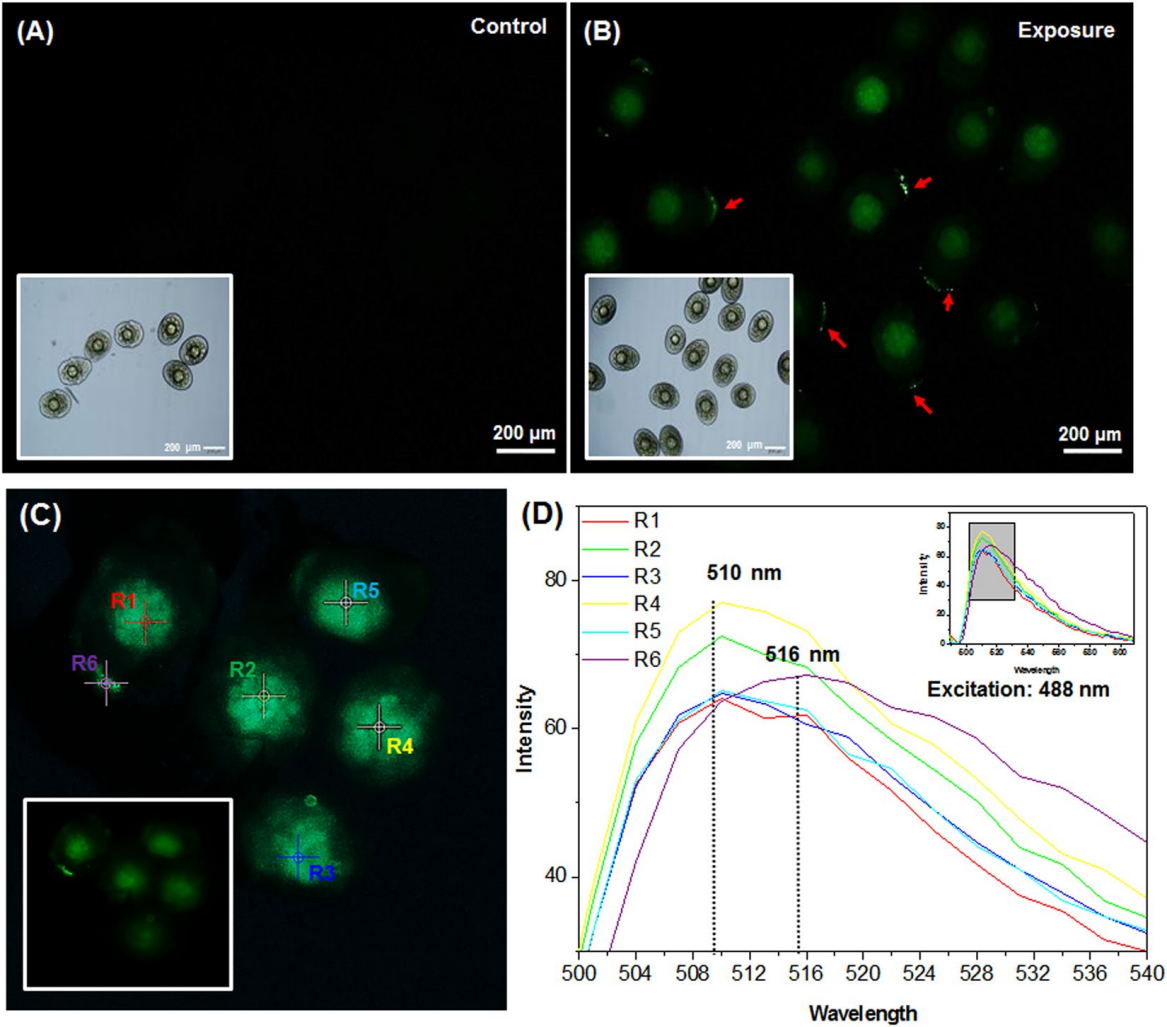
Green fluorescence images of *Daphnia galeata* embryos isolated from the brood chamber of the parent generation. Compared with (**A**) the control group, (**B**) the exposed group shows stronger green fluorescence in the embryo cores, and attached particles (red arrows) on the embryo surface. (**C**) Images from linear unmixing analysis: localization of embryo core (R1–R5) and the attached particles (R6). (**D**) Spectral information of R1–R6: the main emission peak of R1–R5 is at 510 nm, and R6 is at 516 nm.

We also found strong green fluorescence in the embryo cores (Fig. 2C: blue arrows). However, this fluorescence was unexpectedly strong and had a completely spherical shape. To evaluate whether the strong green fluorescence in embryo cores was from PS-NP distribution, we isolated the embryos from the parent generation. The strong green fluorescence was found to be attributed to the PS-NP exposure as the control group showed no fluorescence distribution in the embryo core, whereas the exposed group showed strong green fluorescence (Figs. 3A and B). The attached particles were also observed on the embryo surface (Fig. 3B: red arrows). We performed linear unmixing analyses on the embryo images, which were collected from the emission spectra 489 nm to 609 nm, at an excitation wavelength of 488 nm. The peak of emission spectra of the embryo cores (R1–R5) and the attached particles on the embryo surface (R6) were defined at 510 nm and 516 nm, respectively (Figs. 3C and D).

### Change in lipid storage in *D. galeata* following PS-NP treatment

**Figure 4:**
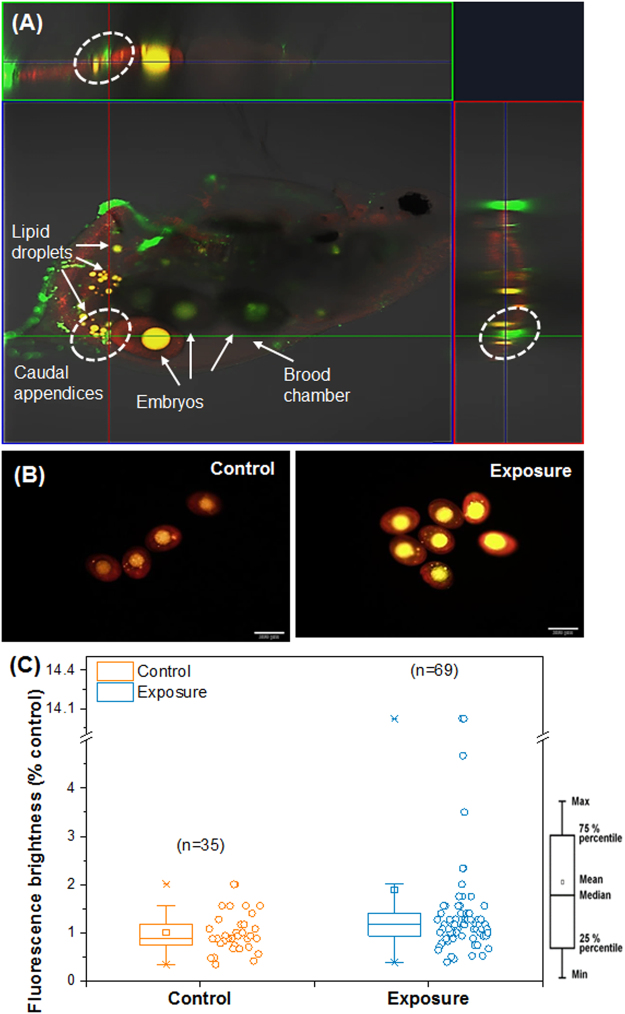
(**A**) Nile Red staining images of *Daphnia galeata* adults. Yellow fluorescence indicates lipid distribution on embryo cores and droplets, and green fluorescence indicates polystyrene nanoparticles (PS-NP) distribution. White circles mark the classification of lipid and PS-NP distribution on caudal appendices. (**B**) Nile Red staining images of control and exposed groups of embryos isolated from the parent generation. (**C**) Quantification of yellow fluorescence brightness in control and exposed groups. Box-whisker plots present the median, minimum, maximum, outliers, and 25^th^ and 75^th^ percentiles of the group data.

The Nile Red staining assay of adult *D. galeata* that had been exposed to PS-NP for 5 days revealed yellow fluorescence, indicating lipid distribution in adult organisms. The lipid droplets and embryo cores were stained by Nile Red, and Z-stack images showed a clear distinction between yellow (lipid) and green (PS-NP) fluorescence (Fig. 4A, white circle). The embryos were isolated from the parent generation, and Nile Red staining was analyzed. Yellow fluorescence was observed in the embryo cores of both the control and exposed groups, with the latter group showing a greater fluorescent brightness (Fig. 4B). Fluorescence brightness was quantified as a percentage of the control group, and average brightness of the exposed group was determined as 189% (Fig. 4C). Despite the tests having been replicated 69 times, the fluorescence in embryo cores showed a wide margin of error (40 to 1400%). We, therefore, determined that there was no significant difference (*p* < 0.01) between the embryos derived from the control and exposed groups.

**Figure 5:**
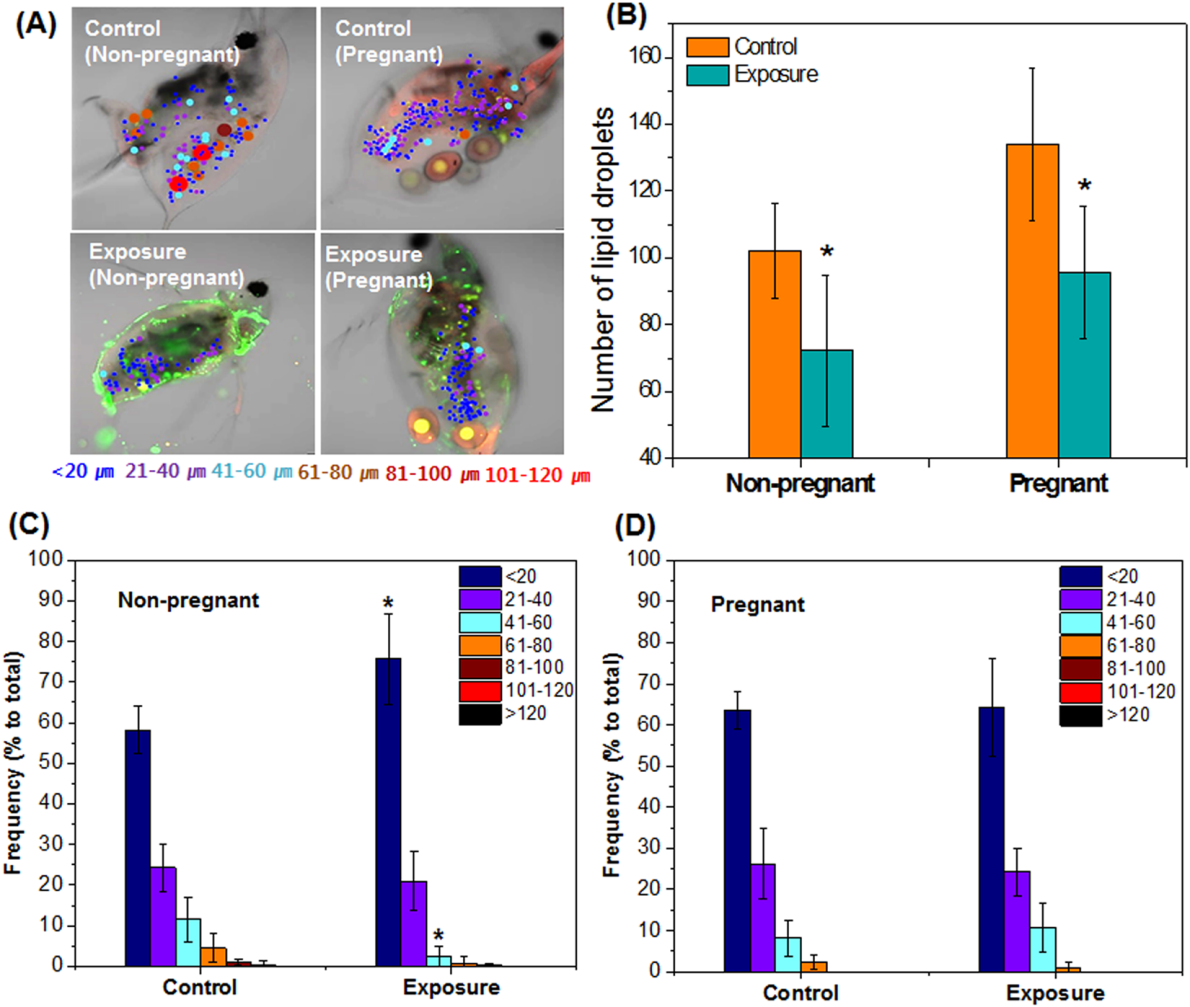
Size distribution of lipid droplets in non-pregnant and pregnant adults of the parent generation. (**A**) Nile Red staining images of different-sized lipid droplets distributed in control and polystyrene nanoparticle-exposed groups. Lipid droplets were classified according to the sizes: <20 μm, 21–40 μm, 41–60 μm, 61–80 μm, 81–100 μm, 101–120 μm, and >120 μm, as represented by various colors. (**B**) The number of lipid droplets per individual. (**C**) The proportion of different-sized lipid droplets per non-pregnant individual, and (**D**) per pregnant individual. Asterisk (*) indicates a significant difference (*p* < 0.05) compared with the control group.

The number of lipid droplets and their size distribution were quantified in adult *D. galeata* after 5 days of PS-NP exposure (Fig. 5A). The number of lipid droplets in the exposed non-pregnant parent generation (70 ± 19 droplets per organism) was significantly different (*p* < 0.05) to that of the control group (102 ± 14 droplets per organism) (Fig. 5B). The size distribution was expressed as a percentage of the total lipid droplets, and the peak droplet size was determined to be <20 μm. The distribution of droplets sized <20 μm was 58 ± 6% in the control group and 76 ± 11% in the exposed group, and droplets sized 21–40 μm was only 12 ± 5% in the control group and 2 ± 3% in the exposed group (Fig. 5C). The number of lipid droplets in the pregnant parent generation was 134 ± 23 and 96 ± 20 per organism in the control and exposed groups, respectively (Fig. 5B). The peak droplet size was also determined to be <20 μm, but no difference in size distribution was observed between the control and exposed groups (Fig. 5D).

Using the same methodology, we evaluated the number of lipid droplets and their size distribution in the offspring generation produced by the parent generation that had been exposure to PS-NP (Supplementary Fig. S5A). The number of lipid droplets per organism in the non-pregnant offspring from individuals from the control and exposed groups were 68 ± 8 and 62 ± 12, respectively (Supplementary Fig. S5B). The peak droplet size was also determined as <20 μm, and there was no significant difference in the size distribution of droplets <20 μm and 21–40 μm (Supplementary Fig. S5C). The number of lipid droplets in pregnant offspring were 57 ± 15 and 54 ± 7 per organism from the control and exposed groups, respectively, and no changes in size distribution were observed (Supplementary Figs. S5B and D).

## Discussion

The survival of *D. galeata* significantly decreased after 2 days of exposure to 5 mg/L of PS-NP, and reproduction was reduced after 5 days of exposure (Figs. 1A and B). Previous studies reported that nano- and micro-sized plastics can induce adverse effects in *Daphnia* species. Besseling *et al*.^19^ reported that a reduction in body size and reproduction occurred in *D. magna* after exposure to 0.22–103 mg/L of nano-sized polystyrene (~70 nm), and Nasser and Lynch^21^ found that *D. magna* secreted protein as a defensive mechanism after exposure to 10 μg/mL of COOH-polystyrene. Short-term exposure to micro-sized plastics can affect survival and mobilization^20,22^, whereby the effective concentration for 50% immobilization (EC50) was 57.43 mg/L of 1-μm polyethylene^22^. We found that embryos in the exposed group showed abnormal development, and hatching rates were dramatically decreased (i.e., by 10 ± 15%). We suggest that there was a relationship between the direct contact of PS-NP with the embryos in the brood chamber and the alterations in the biological development that occurred.

The biodistribution of nano-sized polystyrene in *Daphnia* species has been reported in only two previous studies. Rosenkranz *et al*.^23^ reported that fluorescent-labeled polystyrene was clearly distributed in the gastrointestinal tract, and Nasser and Lynch^21^ found that COOH-polystyrene particle internalization did not occur throughout the gut. We also observed PS-NP fluorescence in the intestine of *D. galeata* and in the thoracic appendices, the brood chamber, and caudal appendices (Fig. 2). Some particles were aggregated with lipid droplets and in the ovary, and they penetrated the brood chamber through the caudal appendices. The potential pathway of PS-NP intake in *Daphnia* species is shown in Fig. S3 (Supplementary Information) and concurs with previous studies of nano-sized particles. Nano-sized particles, such as quantum dots, easily adhered to the carapace and entered the gastrointestinal tract^34^, respiratory tract^35^, and brood chamber^36^. We also found aggregations of PS-NP on the caudal appendices, which is the main route of transfer into the brood chamber, and hence into the embryos. Our data provides visual evidence of this PS-NP transfer, which resulted in some embryonic changes. Embryos in the exposed group showed strong fluorescence in their cores that demonstrated a different emission peak from that of the attached particles on the embryo surface (Figs. 3C and D). The green fluorescence in embryo cores changed shape and disappeared in the course of development from 3 to 54 h (Supplementary Fig. S6). We concluded that there was no PS-NP internalization into the embryo core, and that certain biological mechanisms changed following embryo exposure to PS-NP in the brood chamber.

Embryos in the exposed group showed strong auto-fluorescence in their cores. To evaluate whether the increased auto-fluorescence was induced by changes in lipid storage, we used a Nile Red staining assay in *D. galeata* after 5 days of exposure. This fluorescence staining dye detects lipid droplets in living cells with a fluorescence range from red to yellow-gold^37,38^. Lipid staining and PS-NP particles were clearly seen in the brood chamber and caudal appendices of *D. galeata* adults (Fig. 4A), and increasing brightness of Nile Red fluorescence was observed in the exposed group (Figs. 4B and C). The embryo core contains the post-embryonic yolk and plays a very important role as an energy reserve for respiration and embryonic development^39^. The energy is provided by lipids such as triacylglycerol^34^. Previous studies reported that abnormal embryos, including dormant eggs, had suppressed metabolism and contained high levels of glycerol^40^. In the present study, hatching rates were considerably lower in the exposed group (Fig. 1D), a phenomenon that may be related to changes in lipid storage due to PS-NP exposure in the brood chamber. Many previous studies have reported evidence of micro- and nano-sized plastics toxicity in *Daphnia* species^19–23^, but this is the first report of the changes in lipid storage in embryos following exposure to plastics in the brood chamber.

We also quantified the number of lipid droplets in adult *D. galeata* and noted a significant decrease (*p* < 0.05) in both pregnant and non-pregnant adults that were exposed (Fig. 5B). Lipid storage in *Daphnia* occurs mainly in the form of spherical lipid droplets^41,42^. Changes in the accumulation of lipids is a sensitive parameter that indicates that organisms may be experiencing adverse conditions such as toxicity and/or starvation^43^. Previous studies found that disturbances in lipid metabolism can be induced by plastic exposure^29,30^. It is likely that the lipid storage distribution observed in *D. galeata* in this study was induced by the PS-NP treatment. This effect was not continued in further generations, and the offspring of both control and exposed groups showed similar number of lipid droplets (Supplementary Fig. S5B). According to previous studies, an increase in the size of the lipid droplets in *Daphnia* indicates chemical stress^43,44^. However, in the present study, we found that the <20 μm sized lipid droplets increased simultaneously with a decrease in the 21–40 μm sized lipid droplets in the non-pregnant exposed individuals (Fig. 5C). Lipid droplets consist of neutral lipids (triacylglycerol and cholesterylesters) surrounded by phospholipid and cholesterol^45^, and many previous studies reported that the number and size of lipid droplets can be changed by cyclic storage of triacylglycerol during the reproductive cycle^41,42^. Lipids make a major contribution to embryo production in crustaceans^45,46^, which may explain the significant decrease in pregnant individuals in the PS-NP exposed group (Fig. 1B). These effects did not persist in the pregnant *D. galeata*, because the number of embryos and size distribution of lipid droplets did not show any significant difference (Figs. 1C and 5D, respectively). There were similar numbers of lipid droplets in the offspring of individuals in the control and exposed groups, (Supplementary Fig. S5B), and there was no significant difference in the distribution of lipid droplet size between groups (Supplementary Figs. S5C and D). The offspring were not exposed to PS-NP upon hatching and did not show the adverse effects that were apparent in the parent generation.

These results represent an initial foray into the study of multigenerational effects of polystyrene. Future studies will be necessary to confirm the responses of the second and third generation, and the mechanisms behind these effects should also be investigated.

We performed a toxicity assay using *D. galeata*, and determined the potential pathways of PS-NP transfer and its effect on the behavior of this species. PS-NP accumulated and penetrated the thoracic appendices, lipid droplets, the ovary, caudal appendices, and the brood chamber, but no internalization into embryos occurred. Although the embryos did not internalize the PS-NP, they were directly exposed to penetrated particles in the brood chamber through the caudal appendices. Some embryos showed a high level of lipid storage in response to an abnormal state such as a dormant egg, and a significant increase in abnormal embryo development and mortality was observed in the exposed group. The number and size of lipid droplets in exposed *D. galeata* adults changed before pregnancy. We propose that this phenomenon is related to changes in cyclic lipid storage during PS-NP exposure, which may explain the significant decrease in reproduction in the exposed group. After embryo provisioning, the number and size of lipid droplets in *D. galeata* adults recovered to a level that was similar to that of the control group, and the number of embryos in the brood chamber showed no significant difference. PS-NP seems to reduce fertility through changes in lipid storage during the reproductive cycle, an influence that does not extend beyond embryo production. Our study provides visual evidence of PS-NP transfer in a *Daphnia* species and suggests that there is a relationship between PS-NP toxicity and lipid storage. We found that nano-sized plastic particles can induce disturbances or changes of lipid storage in freshwater *Daphnia* species, resulting in adverse effects on the embryonic and reproductive developmental stages. Further investigations are needed to understand the mechanisms that are affected when freshwater organisms are exposed to nano- and micro-sized plastics.

## Methods

### Target materials and organisms

PS-NP were purchased from Bangs Laboratories, Inc. (Fishers, IN, USA). The particles were dispersed in deionized water with 0.1% Tween 20 and 2 mM of NaN_3_. and were tagged with green fluorescence with a 480 nm excitation and 520 nm emission. According to the manufacturer, the mean diameter of each particle is 51 nm, and the particles meet the official primary particle standards from National institute of Standards and Technology. The actual size of PS-NP was determined as 52 ± 5 nm (*n* = 134) using Field emission transmission electronic microscopy (FE-TEM; JEM 2100 F; Jeol, Japan). The MHW (NaHCO_3_, 96 mg/L; KCl, 4 mg/L; MaSO_4_, 60 mg/L; CaSO_4_ 0.5H_2_O, 78.8 mg/L), was analyzed via the light scattering technique (Zetasizer Nano ZS, Malvern Instruments, UK). The average PS-NP hydrodynamic diameter and zeta-potential were determined as 57.45 nm and −17.4 MHW, respectively.

*D. galeata* specimens were collected from Ilgam Lake (127.0761 N, 37.5405 E, Seoul, South Korea). The culture was maintained in the laboratory for 5 years at 21 °C with a 16:8 h light cycle in MHW, and was supplied a mixture of green algae (*Chlamydomonas reinhardtii* and *Chlorella vulgaris*) as a food source every day. Juvenile *D. galeata* (approximate age: 24 h) were collected for the PS-NP toxicity assay and cultured under the same above-mentioned conditions.

### Test design

Preliminary series tests were conducted to evaluate the effects of PS-NP on *D. galeata* adults and embryo development. A test concentration of 5 mg/L of PS-NP was selected because preliminary tests showed that it was the highest concentration that caused negligible effects on embryo formation in *D. galeata* (data not shown). A 50-mL volume containing PS-NP was prepared in MHW, and 5 *D. galeata* juveniles (<24 h) were exposed for 5 days, with 3 replicates. *D. galeata* were moved to fresh test medium every two days, and green alga cell suspension (*C. reinhardtii*) was provided daily as a food source. Survivors and immobile individuals were counted every day, and the numbers of pregnant organisms and embryos were recorded after 5 days.

To perform the embryo assay, we modified the test method of LeBlanc *et al*.^47^ and Kast-Hutcheson *et al*.^48^ Embryos in the second developmental stage (gastrulation) were isolated from the parent generation after 5 days of exposure. The pregnant adults were prepared on a glass slide with a small quantity of MHW, and using two 25 G needles, embryos were carefully removed from the brood chamber. The isolated embryos were washed using MHW and placed into 96-well plates with 200 μL clean MHW. After 3 days, embryo survival and abnormalities (in the antennae, eye, rostrum, heart, carapace, post-abdominal claw, Malpighian tubule, sensory bristles, and tail spine) were recorded daily using a stereoscopic microscope.

### Biodistribution of PS-NP in *D. galeata* adults and embryos

*D. galeata* adults were washed with MHW after 5 days of exposure to PS-NP and placed on hole-slide glass (10127103 P, CITOGLAS, China) with a small quantity of MHW, with 2–3 drops of 5% agar solution added. The distribution of PS-NP in *D. galeata* was determined using optical fluorescence microscopy and confocal laser scanning microscopy (CLSM, LSM710, Carl Zeiss, USA). The optical fluorescence microscopy was equipped with a fluorescent filter (excitation, 460–495 nm; emission, 510 nm; U-MWIB3, Olympus Cooperation, Tokyo, Japan). For CLSM analysis, PS-NP were excited with an Ar-laser (458 nm, 25 mV), and the emission spectra were collected at 489–609 nm. Z-stack images for *D. galeata* adults were collected from 398–468 slices covering a depth of approximately 1 μm and an area of 1,350 × 1,350 μm^2^. The 2.5D image analysis, which provided bright/fluorescence-field images of distribution versus intensity signal, was performed, and the linear unmixing analysis was investigated to determine the spectral information for PS-NP on *D. galeata* adults and embryos. The linear unmixing analysis plotted the pixel intensity versus the emission spectra, which enabled us to collect the emission spectral profile (489–609 nm) of the localized points. All the images were analyzed using ZEN software.

### Estimation of lipid storage in *D. galeata* adults and embryos

To estimate the effects of PS-NP on lipid storages in *D. galeata* adults and embryos, Nile Red (9-diethylamino-5H-benzo[a]phenoxazine-5-one) staining assay was used. The samples were washed once using clean MHW medium before staining, and the stock solutions of Nile Red (250 μg/mL in DMSO) were diluted to 1 μg/mL using MHW. *D. galeata* adults and embryos were transferred in Nile Red solution and incubated in darkness for 30 minutes at 20 °C. After staining, the organisms were transferred to clean MHW and the Nile Red solution was removed from the surface. Changes in lipid storage in *D. galeata* adults and embryos were observed using optical fluorescence microscopy and CLSM. The analysis methods were the same as described above. The brightness of fluorescence in embryos was quantified, and Z-stack images of *D. galeata* adults were used to determine the number and size of lipid droplets. To reduce the errors from phase difference of images, 3–4 images of z-axis slices were collected (Supplementary Fig. S7). The number of lipid droplets was counted, and droplets were classified according to the sizes: <20 μm, 21–40 μm, 41–60 μm, 61–80 μm, 81–100 μm, 101–120 μm, and >120 μm. The fluorescence brightness, lipid size determination, and lipid number were measured using ZEN software.

### Data analysis

One-way analysis of variance (ANOVA, *p* < 0.05), followed by a Tukey test were used to test for significant effects of PS-NP on *D. galeata* adults and embryos.

## Electronic supplementary material

Supplementary Information
