# Supplementary material for: Polystyrene nanoplastics inhibit reproduction and induce abnormal embryonic development in the freshwater crustacean Daphnia galeata
Source: Sci Rep. 2017 Sep 21;7:12095. doi: 10.1038/s41598-017-12299-2 (PMC5608696; doi:10.1038/s41598-017-12299-2)
Supplement: Supplementary file 1 — Supplementary Information [file 41598_2017_12299_MOESM1_ESM.pdf]

Supplementary Information

Polystyrene nanoplastics inhibit reproduction and induce abnormal embryonic development in the freshwater crustacean *Daphnia galeata*

Rongxue Cui[1], Shin Woong Kim[1], and Youn-Joo An[*]

Department of Environmental Health Science, Konkuk University, 120 Neungdong-ro, Gwangjin-gu, Seoul 05029, Korea

[1] These authors contributed equally.

* Corresponding author: Youn-Joo An
Tel.: +82 2 2049 6090
Fax: +82 2 2201 6295
E-mail: anyjoo@konkuk.ac.kr

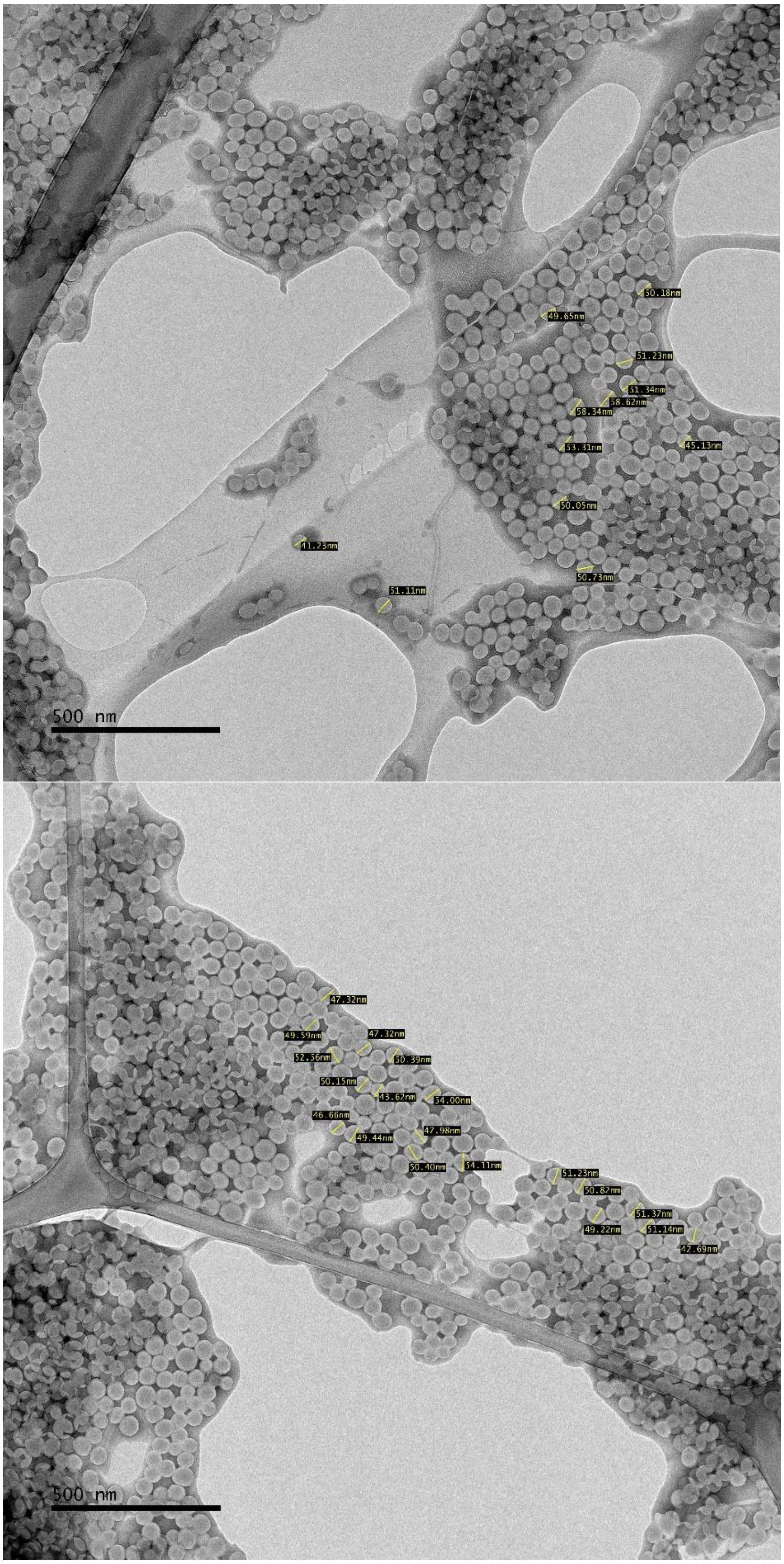

Figure S1. TEM images of polystyrene nanoparticle (PS-NP)

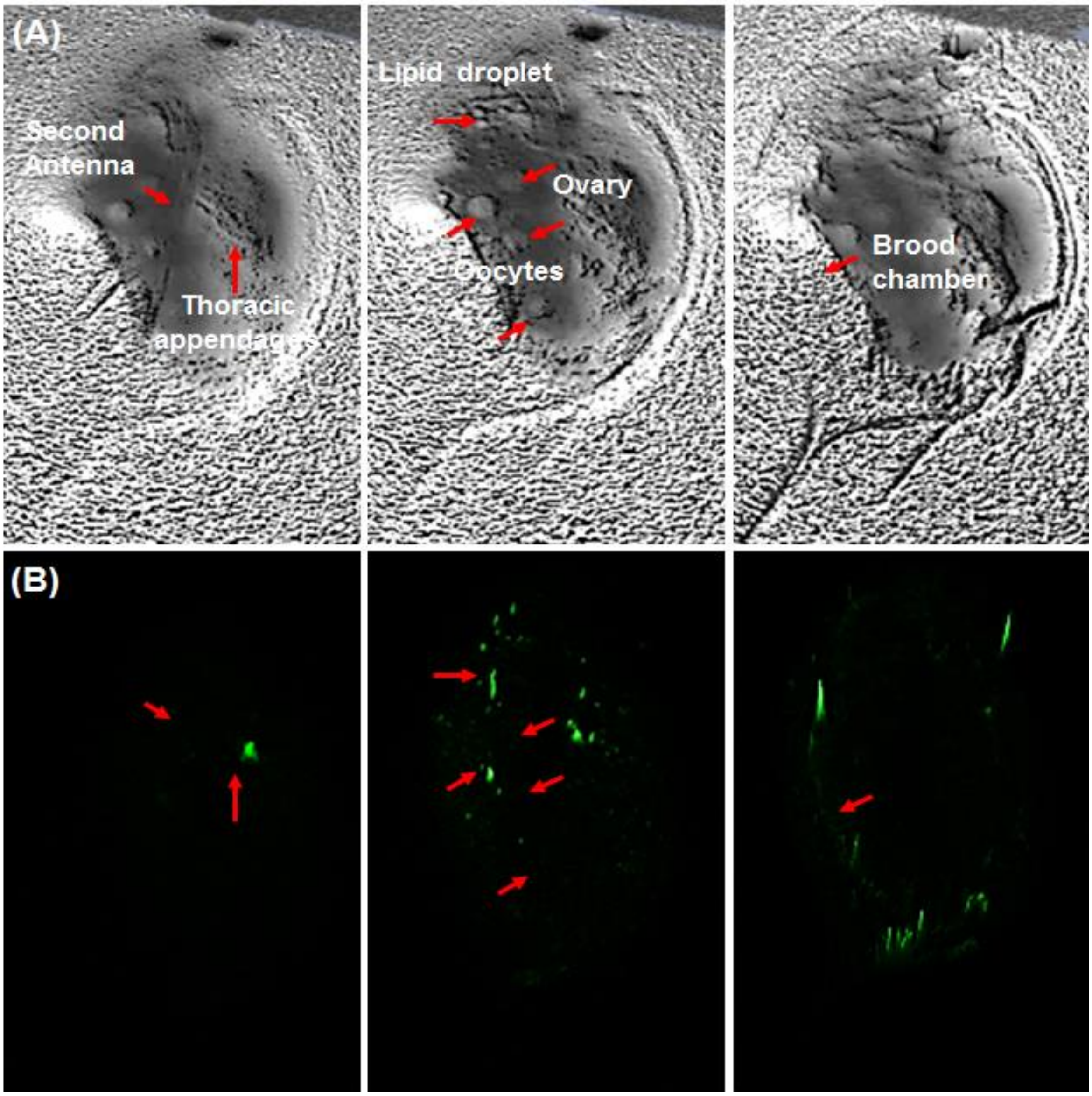

Figure S2. 2.5D images from the confocal laser scanning microscopy of an adult *Daphnia galeata*. (A) Bright-field images show the morphology of thoracic appendages, ovary, oocytes, brood chamber, and lipid droplets. (B) Green fluorescence versus intensity images show the biodistribution of polystyrene nanoparticles. Red arrows in (B) indicate the various *D. galeata* organs indicated in (A).

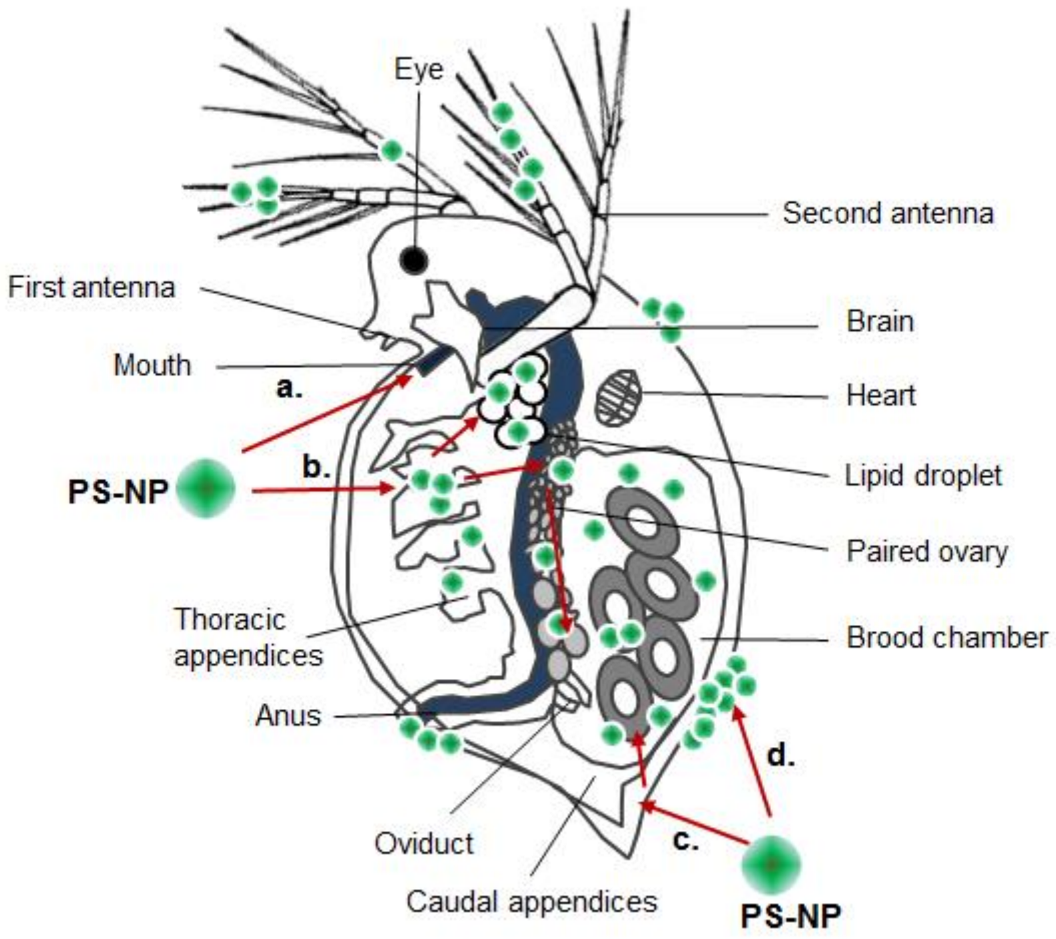

Figure S3. Illustration of the transfer and biodistribution of polystyrene nanoparticles (PS-NP) in *Daphnia galeata*. Various PS-NP intake pathways are shown: a) through the mouth, b) filtration by thoracic appendices, c) penetration into brood chamber through caudal appendices, and d) adsorption through the surface. PS-NP can be transferred into inner lipid droplets or paired ovaries via pathway b).

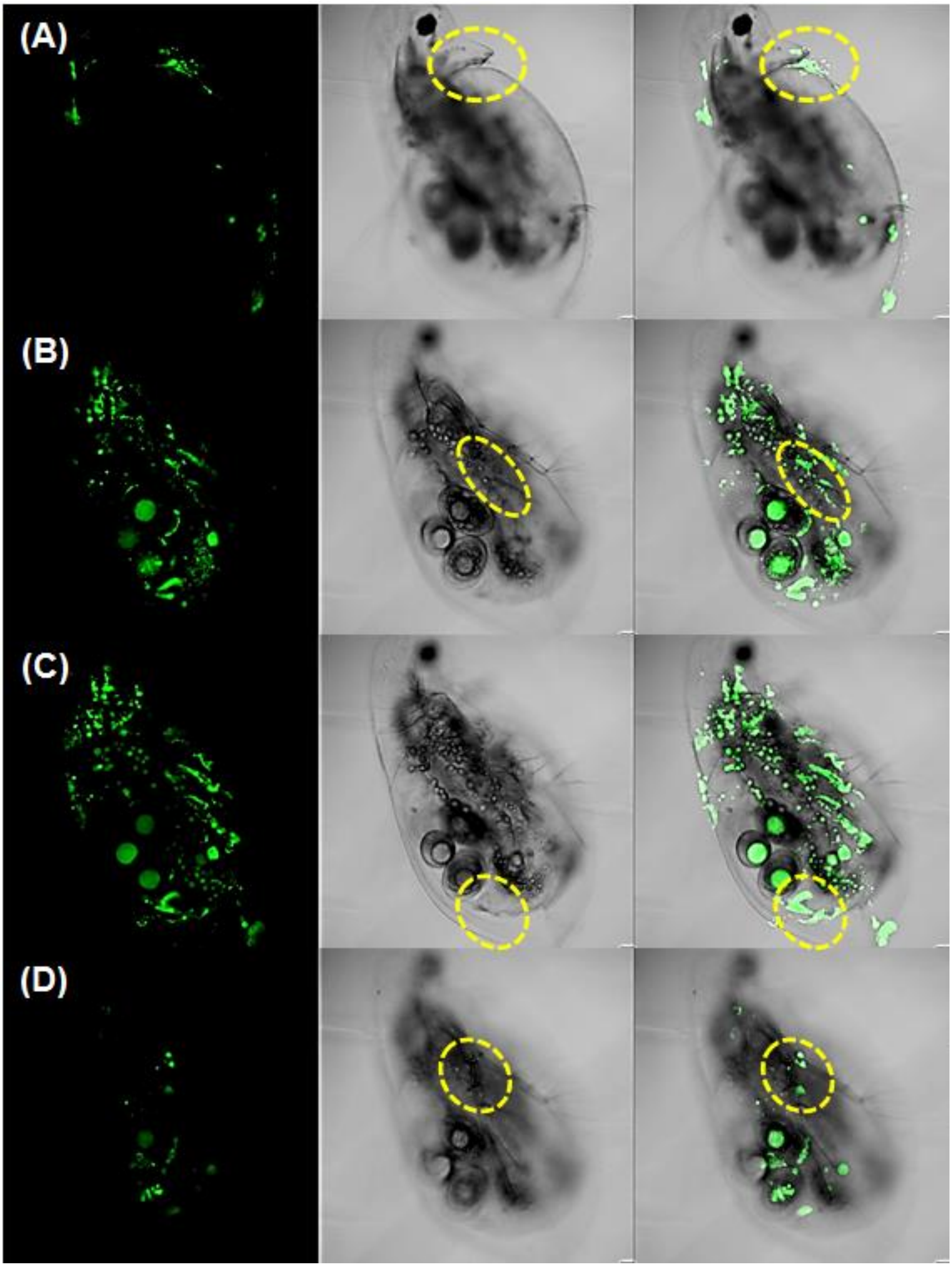

Figure S4. Confocal laser scanning microscopic imaging of polystyrene nanoparticle (PS-NP)-exposed *Daphnia galeata*. Each image is generated from one *Daphnia* individual with a different point of focus. (A) to (D) present visual evidence of various PS-NP intake pathways as seen in a) to d) in Figure S2.

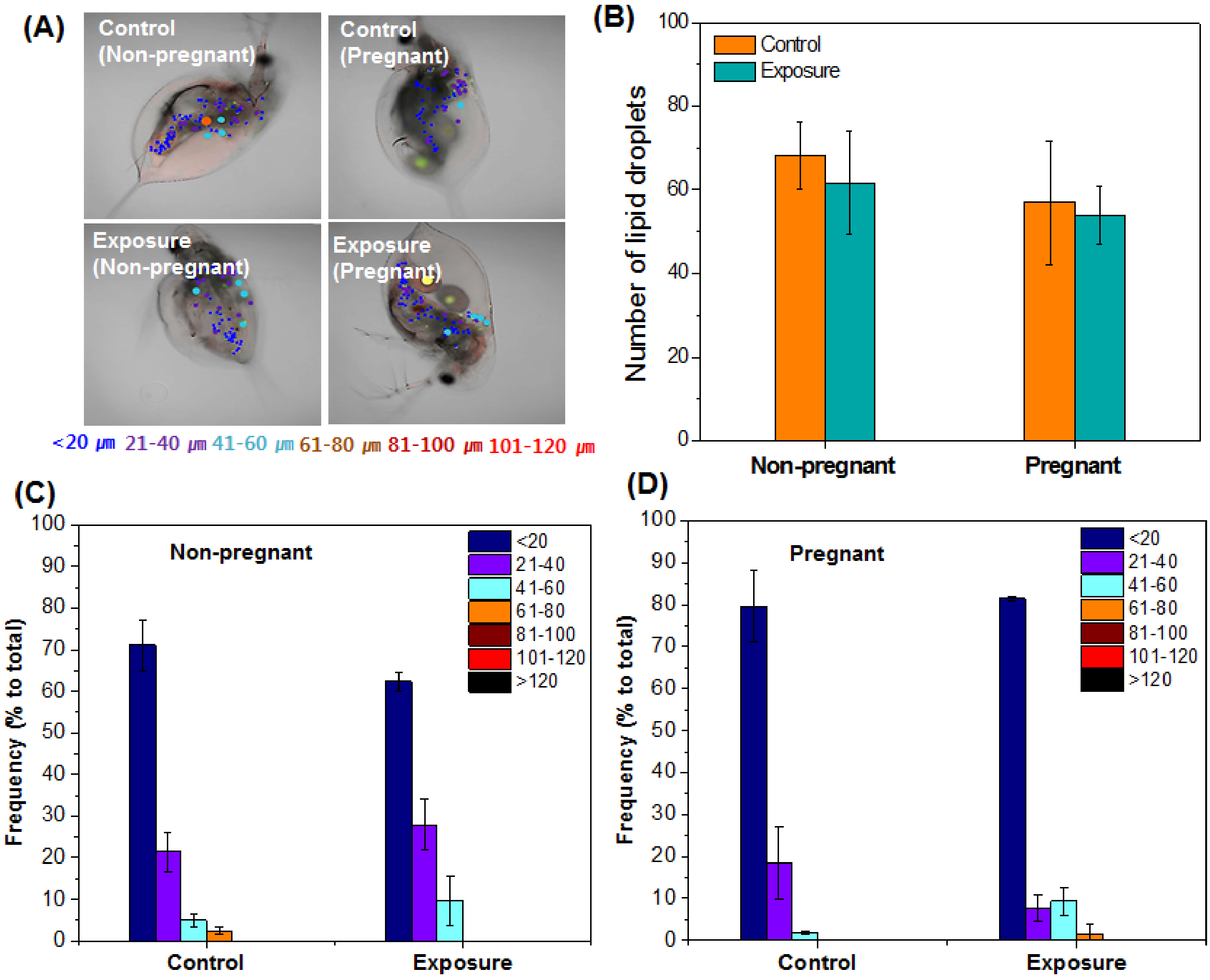

Figure S5. Size distribution of lipid droplets in non-pregnant and pregnant adults of the second generation. (A) Nile Red staining images of different-sized lipid droplets distributed in the control and polystyrene nanoparticle-exposed groups. Lipid droplets were classified according to the sizes: <20 µm, 21-40 µm, 41-60 µm, 61-80 µm, 81-100 µm, 101-120 µm, and >120 µm, as represented by various colors. (B) The number of lipid droplets per individual. (C) The proportion of different-sized lipid droplets per non-pregnant individual, and (D) per pregnant individual.

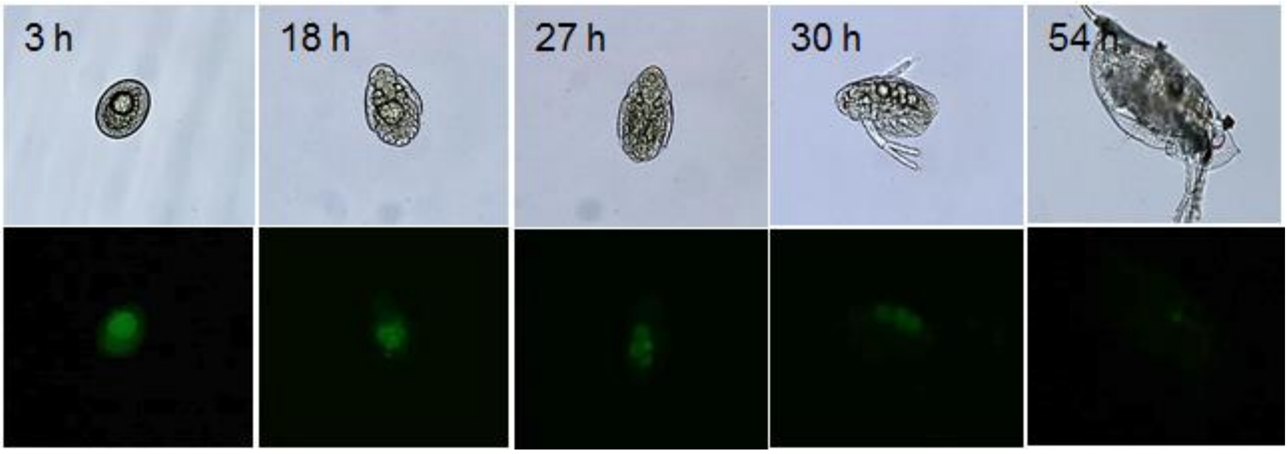

Figure S6. Bright-field and fluorescence images of embryos in the control group. The green fluorescence in embryo cores changed during embryonic development (0–54 h).

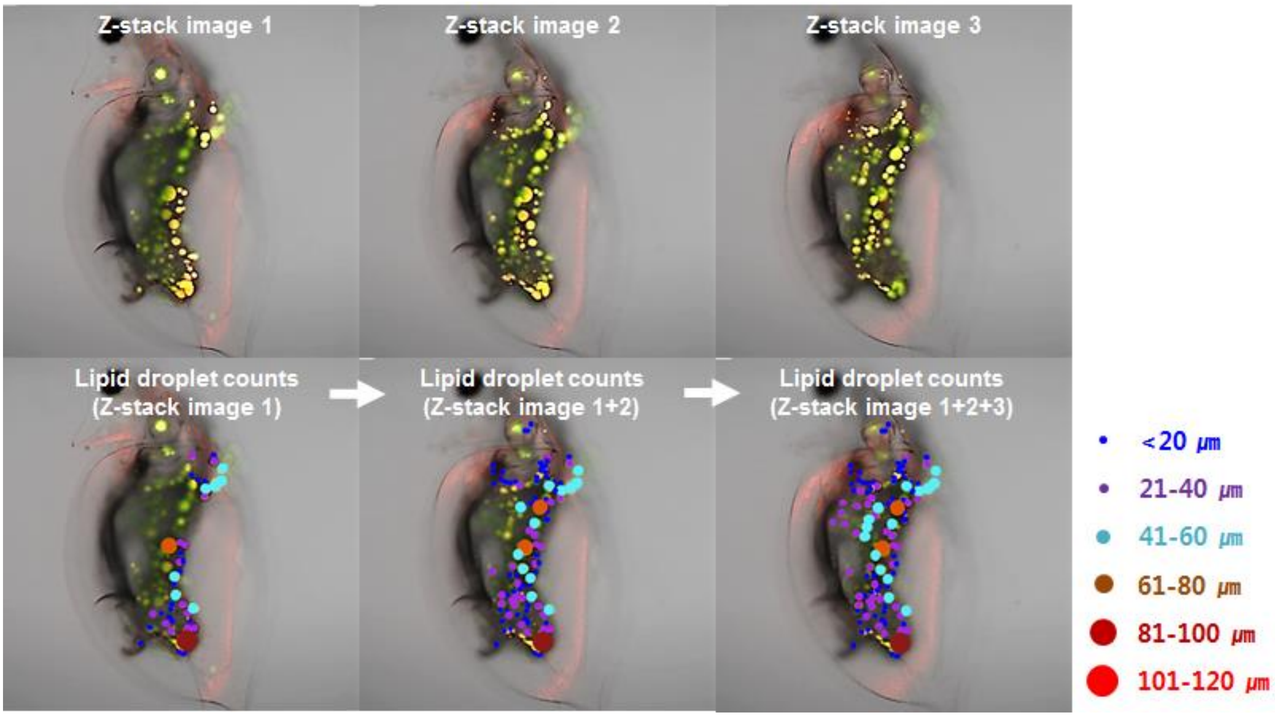

Figure S7. Method example for determining the number and size of lipid droplets. The three images from Z-stack analyses were collected at the different points of focus, and the number of lipid droplets were counted and combined consecutively. All droplets were classified according to the sizes: <20 μm, 21-40 μm, 41-60 μm, 61-80 μm, 81-100 μm, and 101-120 μm.
